# Nutritional management for post-stroke sarcopenia risk and multi-comorbidities patient via percutaneous endoscopic gastrotomy: a case report and review of the literature

**DOI:** 10.3389/fnut.2024.1474328

**Published:** 2024-11-20

**Authors:** Sofia Oliveira, Beatriz Martins, Paula Pereira, Maria Leonor Silva

**Affiliations:** ^1^Nutrition Sciences Student, Egas Moniz Center for Interdisciplinar Research (CiiEM), Egas Moniz School of Health & Science, Caparica, Portugal; ^2^H&TRC-Health and Technology Research Center, Lisbon, Portugal; ^3^Nutrition Lab, Applied Nutrition Research Group, Egas Moniz Center for Interdisciplinar Research (CiiEM), Egas Moniz School of Health & Science, Caparica, Portugal

**Keywords:** post-stroke, malnutrition, dysphagia, sarcopenia, nutritional status, percutaneous endoscopic gastrostomy

## Abstract

Stroke is a major cause of morbidity and mortality worldwide, often leading to complications such as malnutrition, dysphagia, and sarcopenia. We present the case of a 78-year-old male with a history of ischemic stroke and multiple comorbidities, who was underweight and weakened. Over a 10-month follow-up period, a percutaneous endoscopic gastrostomy (PEG) tube was placed, and nutritional management was carried out based on biochemical and nutritional status assessments. Anthropometric and blood biochemical parameters confirmed the need to adjust protein and energy intake to the patient’s requirements. Personalized nutritional intervention, including a caloric surplus and dietary adjustments, resulted in weight gain, improved muscle mass and biochemical blood parameters. This case report highlights the comprehensive nutritional management of a post-stroke patient to improve outcomes and quality of life.

## Introduction

Stroke is the second-leading cause of death and third-leading cause of death and disability combined worldwide in 2019 (1). According to Wafa et al., the number of people living with stroke in Europe is expected to rise by 27% from 2017 to 2047, due to population aging and improved survival rates (2).

Several risk factors have been associated with stroke, including modifiable and non-modifiable risk factors. The main modifiable risk factor is hypertension, but there are others such as hyperlipidemia, diabetes *mellitus*, smoking, physical inactivity and diet. Age, race and genetics include the main non-modifiable factors.

Stroke can be classified into two main types: ischemic, accounting for about 85% of cases, and hemorrhagic, which makes up around 15% (3). Ischemic stroke occurs due to the abrupt blockage of a blood vessel, which leads an inadequate supply of blood to brain tissue. This can result in irreversible loss of tissue function or cell death (4). Symptoms vary by the affected brain region. When the left hemisphere is affected, common symptoms can include aphasia, right hemiparesis, and right hemianopia; when the right hemisphere is involved, left spatial neglect occurs (3). Post-stroke recovery goes through three phases: the acute phase during the first 2 weeks; the sub-acute phase which occurs for up to 6 months; and the chronic phase which can last for years or a lifetime (5).

Dysphagia is also frequently reported in post-stroke patients (6), with a prevalence of around 42% (7). This condition has been associated with high risk of malnutrition, mortality and additional complications (8). Malnutrition represents another common problem among stroke patients with an estimated prevalence of 20% (9), which can be attributed to dysphagia and can lead to stroke-related sarcopenia (10). Sarcopenia is characterized by the progressive loss of muscle mass, accompanied by a decreased in muscle strength and function (11), and can be influenced by multiple factors, including aging, nutrition, physical activity, and disease. Additionally, in stroke patients, the neurological disorders, inflammation, and inactivity causes changes in skeletal muscle that can also contribute to sarcopenia development (12).

In this context, assessing the nutritional status of stroke patients is essential to ensure the patient’s nutritional needs. Percutaneous endoscopic gastrostomy (PEG) placement has been recommended for post-stroke patients with dysphagia to improve nutritional status and overall well-being (13). The malnutrition interventions in post-stroke patients shows inconsistent results, possibly due to a lack of consensus on tools for identifying this condition in these patients (14). Nutritional supplementation in these patients is only recommended in malnutrition cases (15). However, although there are possible benefits associated with calorie and protein supplementation in these individuals, there is still a lack of evidence to support this intervention (15).

We present a case report of a post-stroke patient with sarcopenia risk and multi-morbidity who was receiving artificially feeding through a PEG tube and the nutritional management strategies implemented to improve his nutritional status and overall quality of life.

## Case description

### Patient diagnosis and clinical history

A 78-year-old male with a history of hypertension, dyslipidemia, vascular epilepsy and a medical diagnosis of acute ischemic stroke was admitted to the nursing home (August, 2023). The stroke resulted in a right-side paralysis with loss of mobility and the ability to eat orally. The patient had dysphagia and a global aphasia, but when stimulated he was able to look and tried to verbalize through imperceptible sounds. Physical examination, showed right-sided hemiparesis in both lower and upper limbs, rendering him fully dependent for all activities of daily living, edema in the right upper limb, muscle spasticity due to increased muscle tone, pulse flexion and ankylosis. He had a nasogastric tube (SNG) placed and was eating a homemade liquid diet (HLD).

The HLD included a smoothie made with semi-skimmed milk, dairy flour and 1 piece of fruit, cooked at breakfast; a vegetable soup with meat or fish (protein enriched soup) and one piece of cooked fruit without sugar for dessert at lunch; at afternoon snack a similar meal to breakfast; and at dinner a similar meal to lunch. Before bedtime the patient also drank a milkshake with semi-skimmed milk and dairy flour as an evening snack.

### Nutritional assessment

The patient’s body weight was estimated using a predictive equation (16). At admission the abnormal anthropometrics values, included an estimated weight of 55.7 Kg, body mass index (BMI) of 18.8 Kg/m^2^ (17), as shown in Table 1. Despite the normal values regarding mid-upper-arm circumference (MUAC) (26.0 cm) and calf circumference (CC) (31.5 cm), the CC value is quite close to the cut-off value for stroke patients (cut-off for CC is <31 cm), so it is possible that the patient could develop sarcopenia (18). Table 1 does not include anthropometric data for the period between September and February, as it was not possible to collect this information due to the patient being at home during that time.

**Table 1:** Anthropometric parameters of patient for 10 months of follow-up.

Anthropometric parameters	1st month (August, 2023)	2rd month (September, 2023)	7th month (February, 2024)	8th month (March, 2024)	9th month (April, 2024)	10th month (May, 2024)
Mid-upper-arm circumference, cm (Normal value <24 cm) (31)	26.0	25.4	22.3	22.5	23.5	24.0
Calf circumference, cm (Normal value <31 cm) (18)	31.5	31	26	27	28	27.8
Estimated weight (Kg) (16)	55.7	53.9	44.4	45.0	48.0	49.6
Body Mass Index, kg/m^2^ (Normal weight 22–27 kg/m^2^) (17)	18.8	18.2	15	15.2	16.2	16.8

The laboratory parameters at the time of nursing home admission and during 10 months of follow-up are summarized in the Table 2. At admission (August, 2023), the abnormal laboratory biochemical parameters included urea (14 mg/dL), creatinine (0.5 mg/dL), hemoglobin (10.9 g/dL), hematocrit (32%) and C-reactive protein (2.09 mg/dL) values.

**Table 2:** Laboratory biochemical parameters of patient for 10 months of follow-up.

Laboratory parameters (normal range)	1st month (August, 2023)	7th Month (February, 2024)	9th month (April, 2024)
Glucose, mg/dL (74–106 mg/dL)	97	184.9	77
Urea, mg/dL (19–49 mg/dL)	14	21	18
Creatinine, mg/dL (0.70–1.30 mg/dL)	0.5	0.8	0.59
Hemoglobin, g/dL (13.7–17.2 g/dL)	10.9	12.2	12.1
Hematocrit, % (40–50%)	32	37.2	35
MCV, fL (83–98 fL)	86	90.2	90
MCH, μg (28–32 μg)	29	29.6	31
MCHC, g/dL (32–36 g/dL)	34	32.8	35
RDW, % (11.6–14.1%)	16	15.8	14.6
Neutrophils (1.50–6.50 × 10^9^/L)	3.10	5.3	2.09
Eosinophils (0.02–0.67 × 10^9^/L)	0.40	0.05	0.08
Basophils (< 0.13 × 10^9^/L)	0.04	0.02	0.01
Lymphocytes (1.10–3.5 × 10^9^/L)	1.32	2.01	1.54
Monocytes (0.21–0.92 × 10^9^/L)	0.52	0.32	0.46
Platelets (170–430 × 10^9^/L)	350	278	198
Sodium, mmol/L (136–145 mmol/L)	136	137	141
Potassium, mmol/L (3.5–5.1 mmol/L)	4.3	4,3	3.4
C-reactive protein, mg/dL (< 0.50 mg/dL)	2.09	0.34	2.53

MCV, Mean Corpuscular Volume; MCH, Mean Corpuscular Hemoglobin; MCHC, Mean Corpuscular Hemoglobin Concentration; RDW, Red Cell Distribution Width.

One month after nursing home admission (September, 2023), a chronic bronchitis was diagnosed and symptoms such as, dyspnea, wheezing and productive cough were observed. The patient showed a loss of 1.8 Kg body weight and BMI of 18.2 Kg/m^2^ was observed. A PEG was placed to improve the patient’s nutritional status while maintaining the HLD.

There was no specific medical information regarding the type or severity of the patient’s dysphagia was provided. However, given the diagnosis, a PEG tube was placed to improve the patient’s overall nutritional status and address existing malnutrition and sarcopenia. The decision to place the PEG over the nasogastric tube (NGT), which was present at admission, was made by a multidisciplinary team and considered the specific case, clinical situation, diagnosis, prognosis, ethical factors, and the family’s wishes. The primary goals were to prevent further weight loss, correct nutritional deficiencies, and enhance the patient’s quality of life related to inadequate oral intake (13).

According to ESPEN guidelines, PEG feeding is recommended when a patient’s nutritional intake is expected to be inadequate for over 2–3 weeks (13). In this case, the patient qualified for PEG placement due to a neurological disorder, specifically a dysphagic state following a cerebrovascular stroke, and the requirement for long-term feeding (over 6 weeks) because of moderate malnutrition (13, 19).

The choice of PEG placement rather than continue with the NGT was based on the NGT’s suitability for only short-term use (up to 4–6 weeks). Therefore, PEG is the preferred access device for patients needing long-term enteral nutrition if suitable (19).

**Figure 1:**
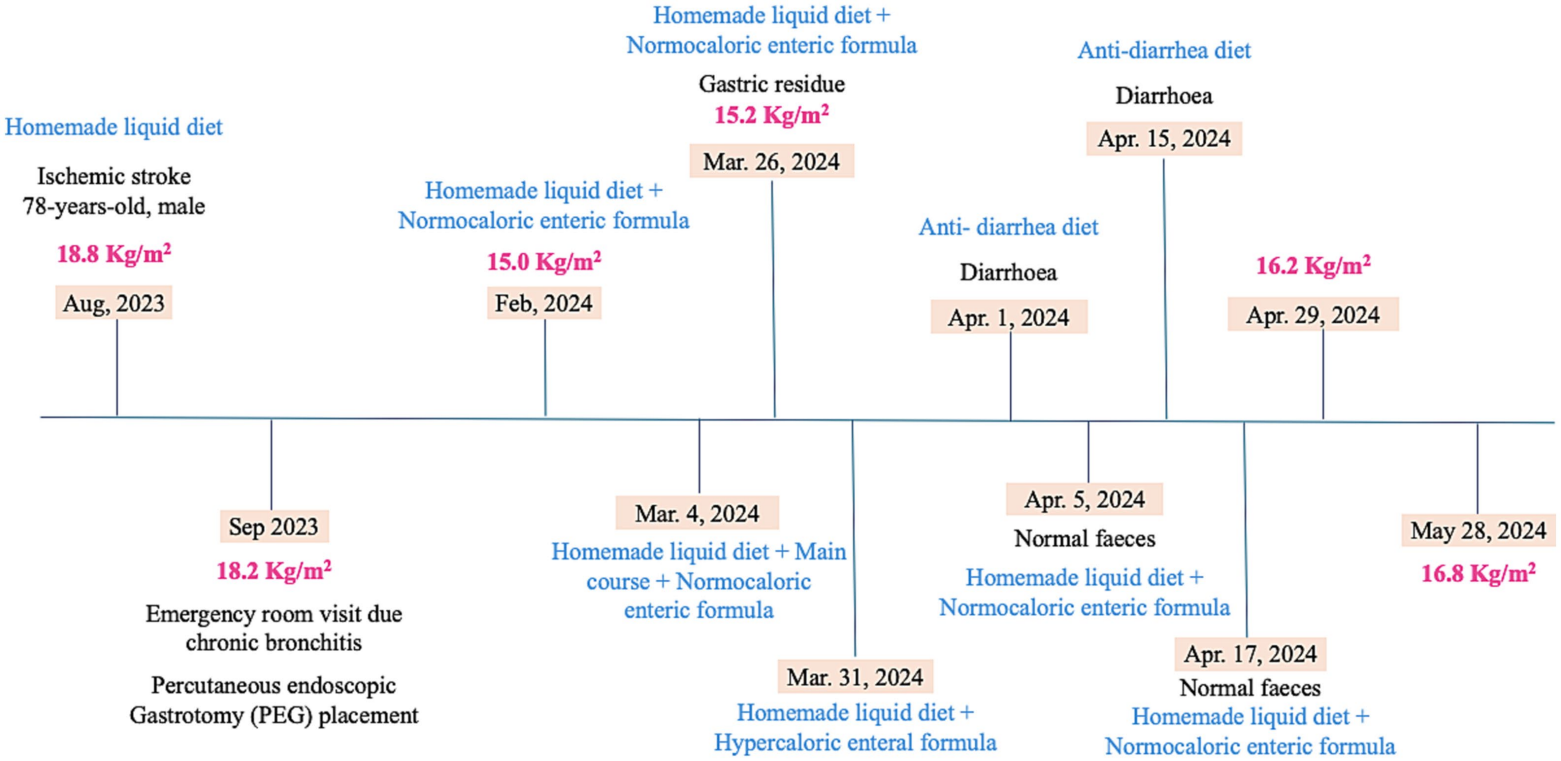
Timeline of nutritional status, dietary parameters and clinical features.

The timeline of the clinical parameters and the nutritional assessment and interventions are shown in Figure 1.

The estimated energy and macronutrient requirements were calculated upon admission to the nursing home, using the Revised Harris-Benedict Equation according to his age, body weight, height, activity factor, organic stress factor and temperature factor. The macronutrient distribution was set to 50% for carbohydrates, 30% for lipids and 20% for protein. The total requirement per day was 1,500 Kcal, 187 g carbohydrate, 75 g protein and 50 g lipids.

### Nutritional management

#### Stage 1: Introduction of normocaloric enteric formula

Six months after PEG placement (February, 2024), a normocaloric enteric formula (NEF) was prescribed, as a complement to the HLD to reinforce the patient’s diet which increased the total energy intake by 500 Kcal daily. The NEF (500 mL per day) was given at breakfast and dinner and the HLD at lunch, afternoon and evening snack. The patient was awake and responsive to stimuli and had no gastric contents before meals, indicating good tolerance to the enteric formula and diet.

#### Stage 2: Main course addition

Despite this, he continued to lose weight, having lost a total of 9.5 kg, which corresponds to a BMI of 15.0 Kg/m2. In this context, to increase daily energy intake (an increase of 227 Kcal), it was added the main course at lunch to the HDL. The main course consisted of meat or fish and potatoes, rice or pasta ground in a blender with a small amount of water to make it liquid enough to pass through the PEG. Table 3 showed the comparison between dietary parameters of nutrition interventions employed to patient for 10 months follow-up.

**Table 3:** Total energy and nutrient composition of the three diets implemented to patient for 10 months of follow-up.

Dietary parameters	Homemade liquid diet	Homemade liquid diet + normocaloric enteral formula	Homemade liquid diet + main course + normocaloric enteral formula	Homemade liquid diet + hipercaloric enteral formula
Energy (kcal)	1,338	1838	2065	2,156
Lipids (g)	35	52	55	64
Saturated fats (g)	15	21	22	24
Monosaturated fats (g)	11	18	19	24
Polinsaturated fats (g)	4	7	8	10
Carbohydrates (g)	168	235	258	287
Sugars (g)	122	128	129	132
Fiber (g)	12	12	14	14
Protein (g)	83	102	128	102
Sodium (mg)	1999	2,399	2,692	2,616
Potassium (mg)	3,338	4,013	4,693	4,611
Calcium (mg)	1,687	2037	2058	2,241
Phosphorus (mg)	1,482	1782	1969	1889
Magnesium (mg)	198	283	316	293
Iron (mg)	10	16	17	18
Zinc (mg)	8	13	15	15
Selenium (μg)	0	35	35	50
Iodine (μg)	170	235	237	284
Vitamin A (μg)	1,100	1,650	1,664	1918
Vitamin D (μg)	6	13	13	17
Tocopherol (mg)	5	13	13	18
Thiamine (mg)	2	2	3	3
Riboflavin ou B2 (mg)	3	3	4	4
Vitamin B6 (mg)	2	3	3	3
Vitamin B12 (μg)	2	4	5	5
Vitamin C (mg)	75	130	144	169
Folate (B9) (μg)	127	272	305	374

At this time (February, 2024), the patient revealed an improvement of previous abnormal biochemical parameters, including, urea (21 mg/dL), creatinine (0.8 mg/dL) and C-reactive protein (0.34 mg/dL). Additionally, the patient revealed an abnormal glucose value which was not altered at admission moment (Table 2).

#### Stage 3: Removal of the main course

The nutritional status was improved slightly 22 days after adding the main course to the diet, in which he gained 0.6 kg of body weight, corresponding to a BMI of 15.7 Kg/m^2^. However, it was observed that the patient maintained the aphasic state and often had gastric contents before meals, which showed a possible gastric stasis. This may have occurred due to a low tolerance to the implemented diet, and in this regard, the main course was removed to avoid over-feeding.

#### Stage 4: Introduction of hypercaloric enteric formula

Taken in account that patient still had a low body weight and visibly weakened state, a hypercaloric enteral formula (HEF) was prescribed to replace the NEF for 5 days. This change of enteric formula allowed the daily calorie intake to increase by 318 Kcal.

#### Stage 5: Antidiarrheal diet

During the following month (April 2024), several episodes of diarrhea occurred, leading to the discontinuation of NEF and implementation of an antidiarrheal diet. The anti-diarrhea diet excludes greens, dairy products and only uses fat-free cooking methods such as grilling and boiling. This diet consists of a dairy-free smoothie made with non-dairy rice flour and an herbal infusion or water for breakfast; a protein-enriched soup without greens and cooked fruit for lunch; a snack similar to breakfast in the afternoon; and a meal similar to dinner at lunch. By the end of month and after recovering diarrhea episodes (normal feces), the patient showed good tolerance to the reintroduction of HLD and the NEF supplementation. It was observed a gain of 3.1 kg in body weight and a corresponding BMI of 16.2 kg/m^2^.

The patient’s biochemical parameters showed an improvement in glucose value; however, abnormal urea (18 mg/dL), creatinine (0.59 mg/dL), potassium and C-reactive protein (2.53 mg/dL) parameters were observed (Table 2).

#### Stage 6: Assessment of nutritional status

One month later (May, 2024), he had gained 1.5 kg in body weight, which corresponds to a BMI of 16.8 Kg/m^2^ showing an improvement in his overall nutritional status since the last anthropometric assessment.

## Discussion

We report a case of a post-stroke patient who received personalized nutritional and dietary recommendations over a period of 10 months, in which adjustments were made to achieve the patient’s nutritional requirements and improve clinical parameters.

To date, few studies have reported the challenges of the nutritional management in post-stroke malnourished individuals with risk of sarcopenia and multi-comorbidities. This case report provides essential insights in the role of personalized dietary management in post-stroke care.

In stroke patients, malnutrition is often associated with sarcopenia, leading to an increased risk of mortality, complications and poor functional outcomes (11). According to Siotto et al., post-stroke patients diagnosed with sarcopenia showed reduced muscle mass and poorer nutritional status, as well as less favorable functional recovery compared to those without sarcopenia (20). Calcaterra et al. found that in 809 stroke patients, the prevalence of sarcopenia ranged from 12.6 to 44.9%. In this study, sarcopenia was associated with the risk of malnutrition, as indicated by Mini Nutritional Assessment (MNA) scores below 24 (21).

Stroke is also associated with several complications, including dysphagia, one of the most common sequelae of acute stroke, which can increase the risk of malnutrition (22, 23). Tagliaferri et al. showed that among a large population of non-institutionalized older outpatients, the risk of dysphagia increased with worsening of the nutritional status, being highest among those who were malnourished (24). Furthermore, Saleedaeng et al. reported that older adults with dysphagia were 4.8 times more likely to experience undernutrition compared to those without dysphagia (25).

Enteric nutrition is essential in the nutritional management for patients with dysphagia following an acute stroke (26). According to Maciejewska et al., dysphagia is a common consequence of ischemic stroke leading to aspiration pneumonia, malnutrition and others adverse clinical complications (27). Early nutritional status assessment is crucial to detect malnutrition and improve post-stroke clinical outcomes (28). In this context, nutritional and dietary adjustments are necessary to ensure the patient’s needs and a PEG or a SNG tube may be required.

Nutritional intervention in post-stroke patients aims to prevent malnutrition and ensure an adequate daily intake of protein and energy (27, 29). On admission, our patient had a BMI of 18.8 Kg/m^2^, which is categorized as underweight according to the Lipchitz classification (17).

CC and MUAC are valid anthropometric measurements to diagnose undernutrition in elderly (30). For this reason, both values were measured periodically (Table 1) to assess the patient’s nutritional status using reference values for elderly individuals. Additionally, although not recommended for diagnosis, MUAC is a valuable screening tool for detecting undernutrition. The MUAC cutoff value is 24 cm and measurements below this indicate underweight (31).

The European Working Group on Sarcopenia in Older People 2 (EWGSOP2) guidelines defines a cutoff value for calf circumference of <31 cm. The CC measurement is used in older adults to predict performance and survival. Thus, when other methods are unavailable, it can serve as a diagnostic tool for muscle mass (18). According to Yang et al., the cutoff value of calf circumference for sarcopenia in stroke patients is <31 cm in male and 30 cm in females. Compared with SARC-F questionnaire and Ishii’s score, calf circumference has the optimal performance in screening stroke-related sarcopenia (32). In the first (August, 2023) and second measurements (Setember, 2023), CC values were ≥ 31 cm, so patient was not yet classified as sarcopenic. However, in the third measurement (February, 2024), the CC value was <31 cm, which confirmed a diagnosis of sarcopenia.

In the chronic phase after stroke, there is still a residual inflammation that might potentially impact on the long-term outcome of stroke patients (33).

According to the Global Leadership Initiative on Malnutrition (GLIM) criteria, the patient is malnourished since he has 1 phenotypic criterion and 1 etiological criterion for malnutrition diagnosis (34). The unintentional weight loss, reduced MUAC and low BMI were categorized as phenotypic criterion, while chronic inflammation post-stroke was categorized as etiological criterion. Malnutrition can be classified using thresholds for severity grading into stage 1, moderate malnutrition; and stage 2, severe malnutrition. Classification into either stage requires only one phenotypic criterion. In this context, taken in account that patient lost around 20% of body weight over a period longer than 6 months (from August, 2023 to February, 2024), it was possible to confirm that the patient was in stage 1 of malnutrition (moderate malnutrition). During the patient’s nutritional management, several diets and supplementations were implemented to adjust food intake to the patient’s nutritional requirements.

Upon initial admission to the nursing home, the diet was inadequate, since there was a deficit of 162 Kcal compared to the estimated energy needs (1,500 Kcal per day). According to Yoshimura et al. study, in older underweight stroke patients, a moderate energy surplus of 250–500 calories per day, considering factors like age and activity levels, is recommended for a safe and gradual weight gain (35).

A NEF was prescribed in addition to HLD to improve nutritional status, increasing daily calorie intake (surplus of 338 Kcal), which falls within the appropriate caloric excess range. Furthermore, the addition of the main course to the daily diet led to a surplus of 565 Kcal per day, which improved body weight (0.6 kg gain in body weight). Despite the dietary adjustments, the patient remained underweight and weak. In this context, a HEF was prescribed to increase the daily calorie intake (additional supply of 656 kcal per day) and 1 month after (April, 2024), the patient increased body weight (gain of 3.1 kg in body weight).

The following period was marked by several episodes of diarrhea that may have led to impaired intestinal absorption (36). Due to these episodes an anti-diarrheal diet was required. Despite the lower caloric value of the anti-diarrheal diet and diarrhea episodes, here was no body weight loss.

During the 10-month follow-up, the laboratory parameters were monitored. At the time of admission to the nursing home, the patient had abnormal laboratory biochemical parameters that included urea, creatinine, hemoglobin, hematocrit and C-reactive protein (CRP) values. Six months later, there was an improvement in the urea, creatinine and CRP levels, however, abnormal glucose values were detected. In the last laboratory assessment, an improvement was observed in glucose values, nonetheless, there were detected abnormal urea, creatinine, potassium and CRP values. Regarding the abnormal glucose and potassium values observed (Table 2), only one elevated glucose value and one low potassium value were observed, which did not allow any definitive conclusions to be drawn. Consistent abnormal levels or the presence of other symptoms are necessary to make further assessments.

Blood urea nitrogen (BUN) levels can be influenced by several factors, including protein intake, corticosteroid use, dehydration, and gastrointestinal bleeding. Low BUN levels may result from low protein intake or severe kidney disease (37).

The patient initially ingested approximately 8 g/day of excess protein, ensuring an adequate protein intake. All the subsequent diets continued to maintain a surplus of protein. Therefore, is unlikely that the low urea values were due to a low protein intake.

Serum creatinine (SCr) is used as an indicator of muscle mass and can be affected by altered nutritional status and the wasting of skeletal muscle mass (38).

In patients with sarcopenia there is a decrease in several metabolites including urea cycle metabolites, serum creatinine and creatinine kinase (39). According to Peng et al., a lower Blood urea nitrogen and Creatinine ratio (BUN/Cr) ratio was associated with an increased risk of both total stroke and ischemic stroke (37).

BUN and creatinine are often used together to estimate eGFR levels, indicator of renal function. Elderly people tend to have reduced muscle mass, which can lead to an overestimation of renal function (eGFR levels) (37).

The patient had sarcopenia, as evidenced by calf circumference measurement. Therefore, it is possible that the low creatinine and urea values were due to the low muscle mass.

According to World Health Organization (WHO), anemia is defined when an individual has hemoglobin levels below 13.0 g/dL in men. Throughout follow-up, the patient consistently had hemoglobin and hematocrit values below the reference values. It was also possible to classify the patient’s anemia as mild, since it did not exceed the minimum limit of 10.9 g/dL (40). Low hemoglobin and hematocrit values can be indicators of anemia and are strongly associated with poor outcome and higher mortality after acute ischemic stroke (41).

CRP is an acute-phase inflammatory protein that can increase significantly at sites of infection or inflammation, and it is a known predictor for severity and outcome in ischemic stroke (42, 43). Its levels are markedly elevated in conditions such as rheumatoid arthritis, various cardiovascular diseases and infections (42). According to Zhong et al., CRP levels in peripheral blood were significantly higher in epileptic patients compared to healthy controls, indicating a strong association between inflammation and epilepsy (44). This connection suggests that the observed elevated CRP values may be attributed to the underlying vascular epilepsy. Noonan et al. conducted a study to investigate the relationship between peripheral inflammation and depression in elderly stroke survivors 1.5 years after stroke. Although they were unable to prove this relationship, they did provide evidence of a sustained peripheral inflammatory response. This response was characterized by elevated levels of CRP and total white cell count 18 months post-stroke (45). Since there was no medical diagnosis to justify the elevated CRP values, besides vascular epilepsy, and the patients’ blood tests were conducted less than a year after the stroke, this timing might also explain why the CRP levels remained elevated for so long. Although high CRP values may be due to the medical diagnosis of vascular epilepsy or peripheral inflammatory response after stroke, we have no clinical reason to prove this.

Dysphagia, sarcopenia, and malnutrition are closely interrelated and played a critical role in this patient’s case. Post-stroke dysphagia compromised the patient’s ability to eat safely, leading to inadequate nutritional intake and subsequent malnutrition. This malnutrition further exacerbated sarcopenia, as insufficient energy and protein intake contributed to muscle mass and strength loss. Additionally, the patient’s age and comorbidities, including hypertension and vascular epilepsy, further complicated the situation, increasing vulnerability to malnutrition and sarcopenia. Nutritional intervention, including enteral feeding via PEG, was crucial to address the swallowing difficulties, improve the patient’s nutritional status, and prevent the progression of sarcopenia. This case underscores the importance of an integrated approach that considers age, comorbidities, and nutritional needs to optimize recovery and overall health.

In this case study, personalized nutritional intervention was implemented for a post-stroke patient, emphasizing the use of anthropometric and biochemical assessments to tailor protein and energy intake. The significance of the study lies in its demonstration that such individualized nutritional management, which included PEG tube placement and caloric surplus, can lead to significant improvements in body weight, muscle mass, and overall clinical outcomes for post-stroke patients.

Enteric nutrition plays a crucial role in addressing malnutrition in post-stroke, particularly because stroke patients often experience difficulties with oral intake due to dysphagia. Malnutrition can significantly impair recovery, worsen outcomes, and increase the risk of muscle wasting. Enteric nutrition provides essential nutrients directly into the gastrointestinal tract via a tube, ensuring that patients receive adequate calories and macronutrients necessary for healing and rehabilitation. Early initiation of enteric nutrition is especially important to prevent or reverse the effects of malnutrition in post-stroke patients. In this context, further studies should be conducted to compare the effectiveness of different enteral nutrition methods, such as PEG tube, nasogastric tube, and modified oral diets, in post-stroke patients with dysphagia and malnutrition over a long-term period. Additionally, we recommend investigating the progression of sarcopenia, muscle atrophy, nutritional status, and functional recovery outcomes at various stages of stroke in patients with severe dysphagia. Finally, evaluating changes in muscle mass, strength, and rehabilitation progress should also be a focus to better understand the role of protein and caloric surplus in enteral nutrition for preventing sarcopenia in post-stroke patients.

Throughout this case study, several limitations were encountered, particularly financial constraints, limited food resources and protocolised diet within the institution, and challenges related to the implemented nutritional approach, as well as the socioeconomic difficulties of the patient’s family and caregivers. Despite these challenges, early diagnosis of sarcopenia and malnutrition was achieved, and effective nutritional interventions and strategies were developed for the patient. As a result, there was an improvement in anthropometric measurements, leading to a better nutritional status.

Ultimately, regular nutritional assessments and timely dietary adjustments proved critical for improving clinical outcomes and preventing malnutrition in post-stroke patients. Additionally, effective communication among healthcare professionals was essential for providing personalized care, highlighting the importance of a multidisciplinary approach in post-stroke management.

## Data Availability

The original contributions presented in the study are included in the article/supplementary material, further inquiries can be directed to the corresponding author.
